# MiRNAs differentially expressed in skeletal muscle of animals with divergent estimated breeding values for beef tenderness

**DOI:** 10.1186/s12867-018-0118-3

**Published:** 2019-01-03

**Authors:** Berna I. G. Kappeler, Luciana C. A. Regitano, Mirele D. Poleti, Aline S. M. Cesar, Gabriel C. M. Moreira, Gustavo Gasparin, Luiz L. Coutinho

**Affiliations:** 1 Department of Animal Science, “Luiz de Queiroz” College of Agriculture, University of São Paulo, Piracicaba, SP 13418-900 Brazil; 2 Embrapa Pecuária Sudeste, São Carlos, SP 13560-970 Brazil; 3 Department of Veterinary Medicine, Faculty of Animal Science and Food Engineering, University of São Paulo, Pirassununga, SP 13635-900 Brazil

**Keywords:** Beef, *Bos indicus*, bta-miR, MicroRNA, Shear force

## Abstract

**Background:**

MicroRNAs (miRNAs) are small noncoding RNAs of approximately 22 nucleotides, highly conserved among species, which modulate gene expression by cleaving messenger RNA target or inhibiting translation. MiRNAs are involved in the regulation of many processes including cell proliferation, differentiation, neurogenesis, angiogenesis, and apoptosis. Beef tenderness is an organoleptic characteristic of great influence in the acceptance of meat by consumers. Previous studies have shown that collagen level, marbling, apoptosis and proteolysis are among the many factors that affect beef tenderness. Considering that miRNAs can modulate gene expression, this study was designed to identify differentially expressed miRNAs that could be modulating biological processes involved with beef tenderness.

**Results:**

Deep sequence analysis of miRNA libraries from *longissimus thoracis* muscle allowed the identification of 42 novel and 308 known miRNAs. Among the known miRNAs, seven were specifically expressed in skeletal muscle. Differential expression analysis between animals with high (H) and low (L) estimated breeding values for shear force (EBVSF) revealed bta-mir-182 and bta-mir-183 are up-regulated (q value < 0.05) in animals with L EBVSF, and bta-mir-338 is up-regulated in animals with H EBVSF. The number of bovine predicted targets for bta-mir-182, bta-mir-183 and bta-mir-338 were 811, 281 and 222, respectively, which correspond to 1204 unique target genes. Among these, four of them, *MEF2C*, *MAP3K2*, *MTDH* and *TNRC6B* were common targets of the three differentially expressed miRNAs. The functional analysis identified important pathways related to tenderness such as apoptosis and the calpain–calpastatin system.

**Conclusion:**

The results obtained indicate the importance of miRNAs in the regulatory mechanisms that influence muscle proteolysis and meat tenderness and contribute to our better understanding of the role of miRNAs in biological processes associated with beef tenderness.

**Electronic supplementary material:**

The online version of this article (10.1186/s12867-018-0118-3) contains supplementary material, which is available to authorized users.

## Background

MicroRNAs (miRNAs) are small 22 nucleotides endogenous non-coding ribonucleic acids (RNAs) [1] that negatively modulate the expression of genes in plants, animals and virus at a post-transcriptional level through cleavage or translational inhibition [2, 3]. MiRNA sequences are highly conserved among species, from nematode to cattle and humans, a reason why they are of central importance to biology and developmental decisions [3–7]. Increasing evidence indicates that miRNAs play an important regulatory role in several biological processes such as cell proliferation, differentiation, neurogenesis, angiogenesis, and apoptosis as well as epigenetic changes [2, 8].

In animals, they were previously reported to be related to embryonic development and function of skeletal muscle, adipose, mammary and immune tissues [4, 8]. For example, miR-1 and miR-133 are muscle-specific and are involved in the modulation of muscle proliferation [9]; miR-133 increases proliferation of C2C12 myoblasts [9]; miR-486 is an inducer of myoblast differentiation [10], and miR-26a is induced during skeletal muscle regeneration [11]. However, little is known about the role of miRNAs in beef tenderness.

Among the traits of economic value in livestock species, meat quality, specifically beef tenderness, is considered the primary attribute of sensory satisfaction of the beef consumers [12–14]. It is a complex trait with economical importance to the beef industry and has been a major focus of many studies [15, 16].

The present investigation was undertaken to identify differentially expressed miRNAs and functional pathways associated with beef tenderness in Nelore (*Bos indicus* species) cattle. We hypothesized that variation in shear force at 14 days of aging could be associated with the difference in miRNA expression in skeletal muscle. Thus, we sequenced miRNAs from *longissimus thoracis* (LT) muscle of animals with high (H) and low (L) EBV for shear force (SF) values to detect differential expressed miRNAs and to identify putative biological processes associated with beef tenderness.

## Methods

### Animals and phenotype

The population used in this study was previously described in detail by Tizioto et al. [17]. Briefly, a total of 390 Nelore steers, offspring of 34 sires unrelated were used to obtain phenotypic data. The animals were raised at pasture until approximately 23 months of age when they were moved to a feedlot with identical nutrition and handling conditions. The animals were slaughtered at an average age of 25 months and an endpoint of 5 mm of backfat thickness (BFT). Immediately after exsanguination, samples were collected from the *longissimus thoracis* (LT) muscle between the 12th and 13th ribs and frozen in liquid nitrogen until RNA extraction. Measurements of meat tenderness were determined by the Warner–Bratzler shear force (WBSF) in 2.54 cm thick steaks obtained from the same muscle after aging at the 2 °C cold chamber for 24 h, at 7 and 14 days postmortem as described into detail by Carvalho et al. [18]. The WBSF values were calculated as the average of eight cores.

For this study, samples were ranked on estimated breeding values for shear force at 14 days of aging (EBVSF14) calculated from the previous study of our group [19], and we selected 34 animals with either the highest (H, n = 15) or lowest (L, n = 19) EBVSF14 to form the groups that were tested for miRNAs differential expression analysis.

### RNA extraction and small RNAs libraries construction

The total RNA was extracted from 100 mg of frozen LT muscle using the TRIzol reagent (Life Technologies, Carlsbad, CA). RNA integrity (RIN) was verified by Bioanalyzer 2100 (Agilent, Santa Clara, CA, USA) and a minimum threshold of RIN seven was used for library construction. Small RNAs libraries were constructed from 1 μg of total RNA from each of the 34 samples using the Illumina TruSeq small RNA Sample Prep Kit (Illumina Inc., San Diego, CA, USA) according to the manufacturer’s protocol. PCR amplification was performed for 15 cycles. Library quality was determined using the High Sensitivity DNA Chip and an Agilent 2100 Bioanalyzer (Agilent Technologies) and quantified with qPCR with the KAPA Library Quantification kit (KAPA Biosystems, Foster City, CA, USA). The individual libraries were adjusted to 20 pM concentrations; sequencing was performed using a Miseq Reagent Kit v3 for 150 cycles in an Illumina Miseq Sequencing System (Illumina Inc., San Diego, CA, USA). This kit allows the generation of 25 million sequences reads per flow cell.

### MiRNA sequencing data analysis

After sequencing, the Illumina CASAVA v1.8 pipeline was used to generate and de-multiplex the raw fastq sequences. The quality of Illumina deep sequencing data was determined by using the FastQC program (version 0.9.5) [20]. Adapters and low quality reads were trimmed using Cutadapt (version 1.2.1) [21] with the following parameters: − b AATCTCGTATGCCGTCTTCTGCTTGC-O 3-m 17-f fastq-q 24, where − b is the Illumina sequence adapter, -O indicates the minimum number of matching bases necessary to recognize the adapter, -m represents the minimum length of sequence and –q stands for the sequences quality.

Filtered reads were then processed following mirDeep2 analysis pipeline [22]. Sequences were aligned to the UMD3.1 *Bos taurus taurus* reference genome (available at the Ensembl database [http://www.ensembl.org/Bos_taurus/Info/Index/]) using the mapper.pl module. Only alignments with 0 mismatches in the seed region (first 18 nt of a read sequence) of a read mapped to the genome were retained.

### Differentially expressed miRNAs

Initially, miRNAs with zero counts for all samples were removed. Next, the miRNAs presenting a count different from zero in at least 1/5 of the samples were maintained. Read count data was normalized to account for differences in starting RNA quantity and other library effects using the Upper Quartile method [23].

The QuasiSeq package [24] developed in R [25] was used to detect miRNAs differentially expressed. QuasiSeq uses a quasi-likelihood method through which is incorporated uncertainty in the estimated variances during the test for differential expression [24] and also provides a self-tuning approach to shrinking miRNAs dispersion estimates. The QuasiSeq method is built from a generalized linear model and allow fixed effects to be added in the model. The PROC MIXED procedure by SAS [26] was performed to determine significant (p < 0.05) fixed effects and covariates that were used in miRNA differential expression analysis by QuasiSeq package. In the QuasiSeq model, the final age and contemporary group (animals of the same slaughter group, origin and birth year) were fitted as a covariate and fixed effect, respectively. High and Low groups were defined for EBVSF at 14 days of aging and used to select animals, while the phenotype for QuasiSeq analysis was WBSF at 14 days of aging, as adopted by Gonçalves et al. [19]. Thus, it is important to highlight that differentially expressed miRNA were identified as a function of WBSF at 14 days of aging.

We used the q-value method to control the false discovery rate (FDR) at 5% [27], to correct for multiple tests (the total number of miRNA tested in the model).

### Target gene prediction and functional analysis

The prediction of target genes of differentially expressed miRNAs was made using the TargetScan website (http://www.targetscan.org/) and the *B. taurus* species. Only the expressed genes in muscle tissue were maintained, which were measured in a previous RNAseq study of our research group [28].

Gene Ontology analysis and functional annotation of target genes were done to identify significative canonical pathways (p-value < 0.1) from the target genes and also to visualize their networks and by IPA Core Analysis from Ingenuity^®^ Pathway Analysis software (IPA^®^, QIAGEN Redwood City, CA).

## Results

### Animals, phenotype and sequencing data

The animals with extreme values of EBVSF14 were selected to compose the high and low groups for miRNA-sequencing analysis (see “Methods”). The average values of SF at 14 days aging and EBVSF14 for the group with high EBVSF14 (H group; n = 15) and the group with low EBVSF14 (L group; n = 19) were 6.50 kgf/cm^2^ and 0.63; and 2.65 kgf/cm^2^ and − 0.62, respectively. A statistical test of means was performed between H and L groups, and the results indicate that the samples selected were divergent for SF at 24 h, seven and 14 days of aging, as well as EBVSF14, but were not different for intramuscular fat and ribeye area (Table 1).

**Table 1:** Test of means (t-test) between groups for shear force (kgf/cm^2^) at 24 h, seven and 14 days of aging, estimated breeding values at 14 days of aging (EBV), intramuscular fat (IMF, %) and ribeye area (REA, cm^2^)

Group	Shear force			EBV 14 days	IMF	REA
	24 h	7 days	14 days			
High	9.70 ± 1.02	7.62 ± 0.93	6.50 ± 1.49	0.63 ± 0.17	2.20 ± 0.96	60.17 ± 8.30
Low	7.87 ± 1.22	3.23 ± 0.60	2.65 ± 0.53	− 0.62 ± 0.21	2.64 ± 0.75	60.05 ± 7.45
p-value	4.17E−05	1.02E−13	3.35E−08	2.49E−19	0.16	0.97

A total of 40,513,215 reads were obtained from miRNA sequencing analysis. After removing low quality reads, adaptor’s sequences and insufficiently tagged, a total of 33,222,144 reads were ultimately maintained. The distribution of the reads length was from 20 to 25 nucleotides (nt), which is the typical length of the small RNA processed by Dicer.

The alignment of the reads against the *Bos taurus* UMD3.1 reference genome showed that ~ 87% of the reads were correctly mapped by the Bowtie2 algorithm using the miRDeep2 software [22]. The read mapping statistic is shown in Additional file 1.

### Identification of known and novel miRNAs in LT muscle

Novel miRNAs in the bovine LT muscle were identified by a deep sequence of 34 small RNA libraries. Known miRNAs were identified by comparison between the precursor and mature miRNAs annotated in the miRBase database (Version 21). In total, 308 mature miRNAs were identified as known miRNAs, and among them, seven were previously described in the literature as skeletal muscle specific (Table 2). This was considered when the miRNA was expressed at least 20 times more than the average of its expression in the other tissues [29].

**Table 2:** Specific skeletal muscle miRNAs identified in samples of both phenotypic extremes

miRNAs ID	Normalized expression level		Mature sequences
	L group	H group	
bta-miR-486	1,754,084.71	1,306,241.09	uccuguacugagcugccccgagg
bta-miR-133a	575,024.68	400,433.38	uuugguccccuucaaccagcugu
bta-miR-1	417,275.36	306,385.93	uggaauguaaagaaguauguauu
bta-miR-206	15,403.29	10,915.25	uggaauguaaggaagugugugg
bta-miR-133b	14,232.50	9769.81	uuugguccccuucaaccagcu
bta-miR-208b	6463.46	5059.15	auaagacgaacaaaagguuugu
bta-miR-499	3375.83	2323.09	uuaagacuugcagugauguuu

To identify novel miRNAs, we compared the seed sequence from miRNAs found in this study with miRNAs already annotated in miRBase for others species close phylogenetically to *B. indicus*. The seed sequence corresponds to the 2–8 nt region of the 5′ end of the miRNA, and is very important for recognition and silencing of mRNA [30, 31]. The *B. taurus*, *H. sapiens*, *M. musculus*, *S. scrofa*, and *E. caballus* species were chosen according to the database of phylogenetic trees of animal genes, TreeFam (http://treefam.genomics.org.cn/). A total of 42 novel miRNAs was identified in the LT muscle from *B. indicus* (Additional file 2).

### Identification of differentially expressed miRNAs

In order to identify miRNAs differentially expressed in LT muscle of animals with divergent estimated breeding values for beef tenderness the QuasiSeq R package was used. Three miRNAs were differentially expressed between the groups (q value < 0.05). Two miRNAs (bta-mir-182 and bta-mir-183) were up-regulated in the L group compared to the H group, and one miRNA (bta-mir-338) was up-regulated in H group compared to the L group (Table 3).

**Table 3:** Differentially expressed miRNAs between animals of the L and H groups based in estimated breeding values for shear force at 14 days of aging

miRNA	Average normalized counts		q-value
	L group	H group	
bta-miR-182	9.91	3.62	1.67E−06
bta-miR-183	6.97	2.73	0.000116092
bta-miR-338	72.41	45.53	0.000291747

### Target genes prediction and functional pathways

**Fig. 1:**
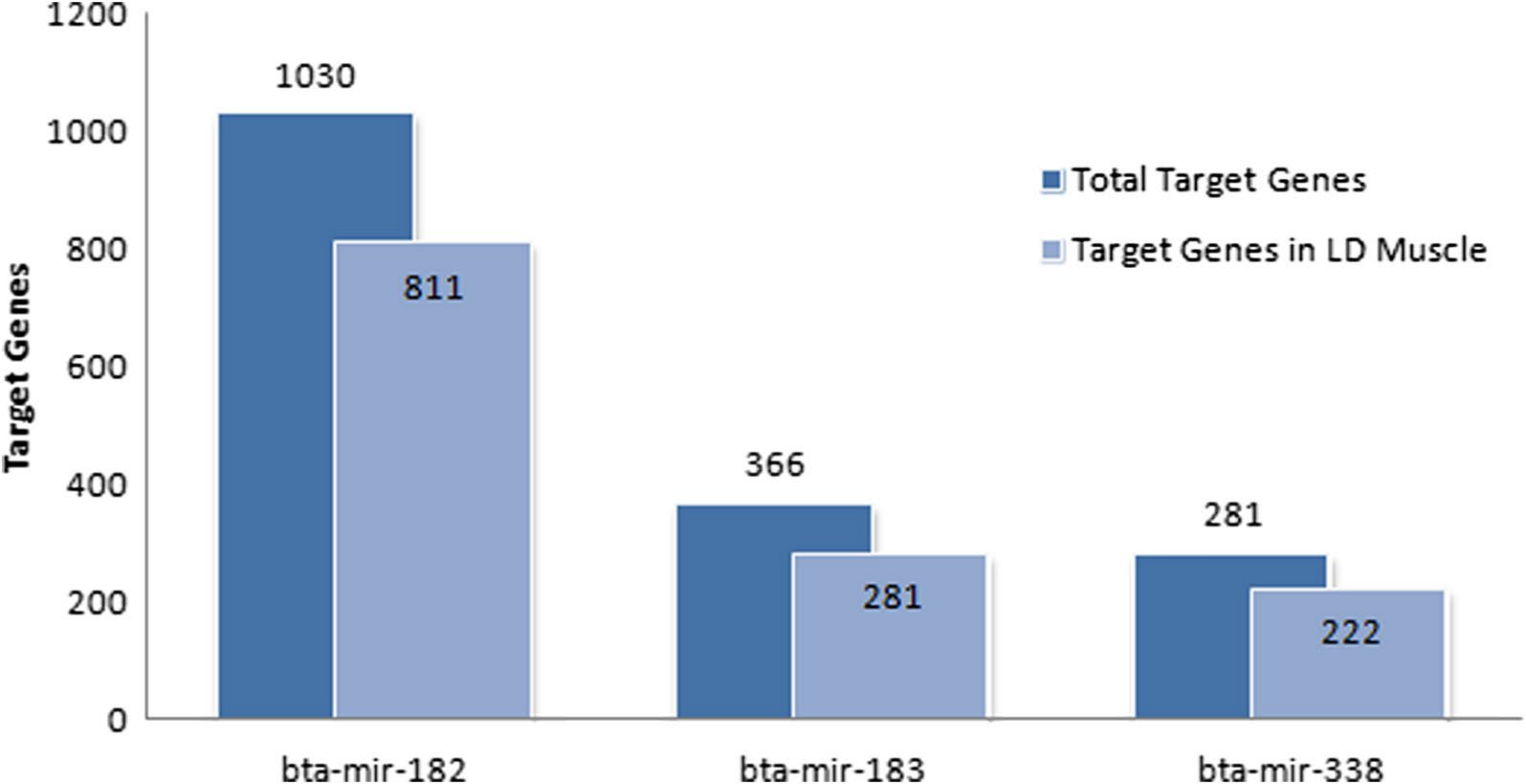
Summary of target genes predicted for bta-mir-182, bta-mir-183 and bta-mir-338 in all tissues and in *Longissimus thoracis* muscle specifically

To identify the potential functions of the differentially expressed miRNAs, bovine target genes of these miRNAs were predicted by TargetScan 6.2 (http://www.targetscan.org/vert_61/). From the list of target genes (Additional file 3), we selected only the genes that are expressed in LT muscle to perform the functional analysis (Fig. 1). For this selection, RNA sequencing (RNA-Seq) data of the same set of samples [28] of this study was used (Additional file 4).

**Fig. 2:**
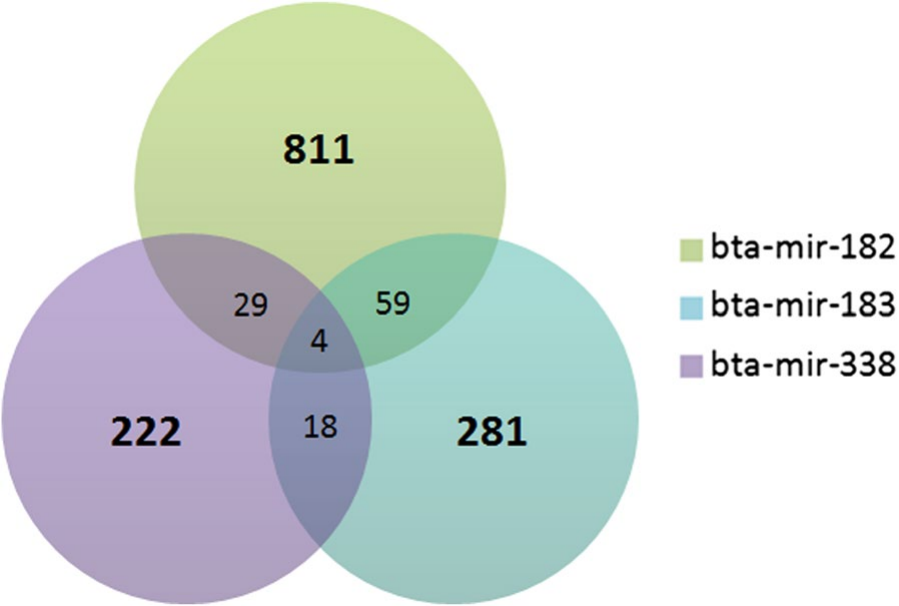
Venn chart of the common and specific target genes of differentially expressed miRNAs

The number of predicted targets expressed in LT muscle for bta-mir-182, bta-mir-183 and bta-mir-338 was 811, 281 and 222, respectively. These numbers correspond to 1204 target genes, after removing duplicates. Four genes (*MEF2C*, *MAP3K2*, *MTDH*, and *TNRC6B*) were found as common targets of the three differentially expressed miRNAs (Fig. 2).

The Ingenuity^®^ Pathway Analysis software conducted the functional enrichment study. First, all predicted target genes were mapped to the IPA Knowledge Base, allowing us to reveal which molecules are being encoded by these genes (Table 4).

**Table 4:** Summary of molecules encoded by target genes predicted of differentially expressed miRNAs

Molecule type	Number of target genes per miRNA		
	bta-mir-182	bta-mir-183	bta-mir-338
Kinase	54	26	17
Transporter	53	19	14
Peptidase	15	5	9
Enzyme	121	36	28
G-protein coupled receptor	11	4	0
Transcription regulator	100	33	31
Translation regulator	14	4	4
Ligand-dependent nuclear receptor	5	3	1
Transmembrane receptor	8	4	7
Growth factor	6	1	1
Ion channel	24	6	3
Phosphatase	19	9	9
Cytokine	3	0	0
Other	378	131	98

**Fig. 3:**
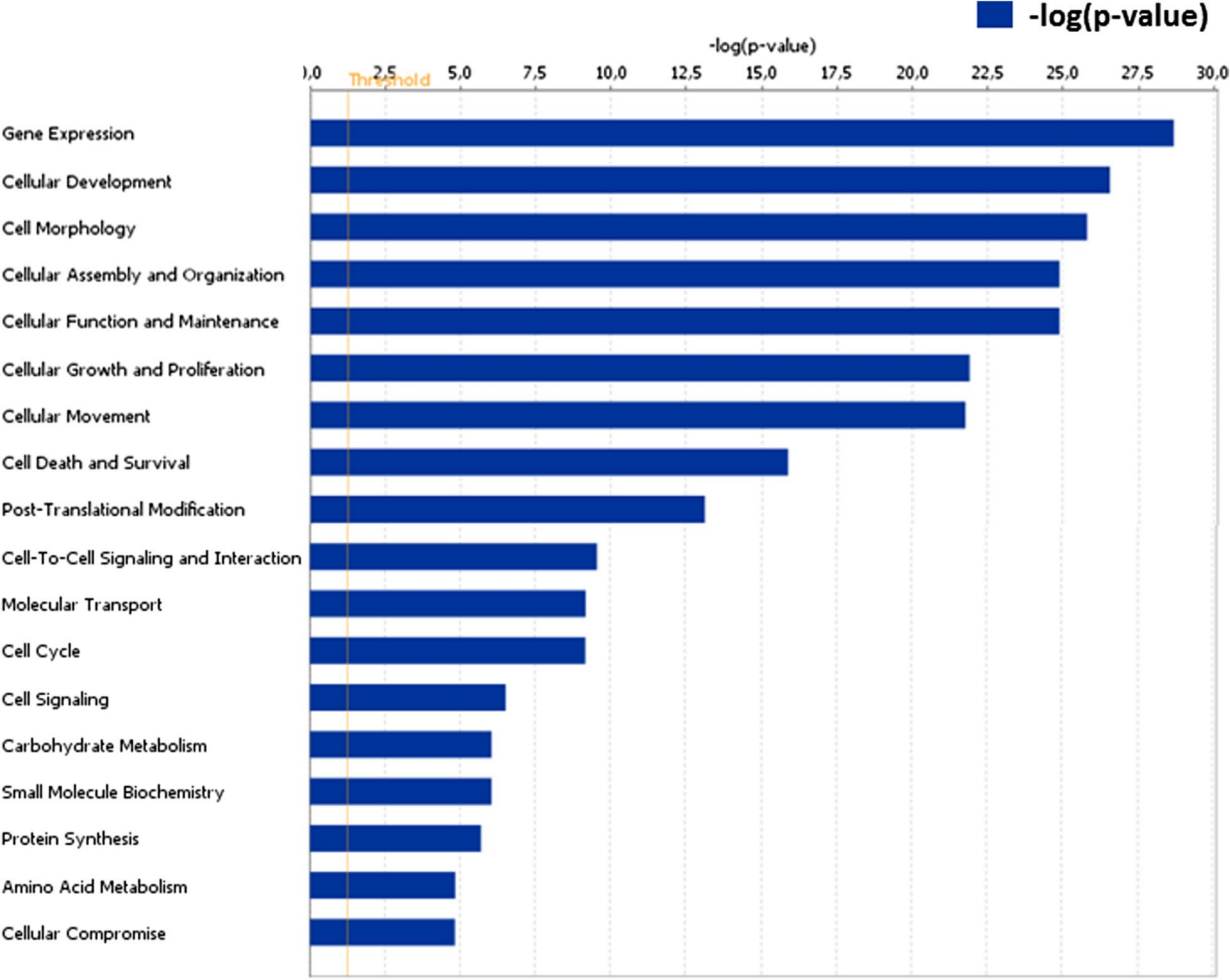
Molecular and cellular functions of total predicted target genes of differentially expressed miRNAs. The likelihood association among the genes and biological functions is represented as − log(p-value), larger bars are more significant than shorter bars considering p-value < 0.05 as cutoff for significance (representing in the threshold vertical line)

Core analysis (functional analysis) revealed that gene expression, cellular development, cell morphology, cellular assembly and organization and cellular function and maintenance were ranked in the top of significant molecular and cellular functions (p < 0.05) (Fig. 3).

The most significant biological processes associated with the target genes for the three differentially expressed miRNAs were cancer, post-translational modification, cell morphology, small molecule biochemistry, nucleic acid metabolism and vitamin and mineral metabolism (Table 5).

**Table 5:** Biological processes related to predicted targets genes

ID^a^	Score	Focus molecules	Top diseases and functions
1	50	35	Cancer, organismal injury and abnormalities, reproductive system disease
2	42	32	Post-translational modification, cell morphology, cellular movement
3	37	30	Carbohydrate metabolism, small molecule biochemistry, hereditary disorder
4	37	30	Post-translational modification, dermatological diseases and conditions, inflammatory disease
5	37	31	Nucleic acid metabolism, small molecule biochemistry, vitamin and mineral metabolism
6	37	30	Cell-to-cell signaling and interaction, nervous system development and function, cell cycle
7	35	29	Developmental disorder, hereditary disorder, neurological disease
8	35	29	Cellular function and maintenance, lipid metabolism, small molecule biochemistry
9	35	29	Cell-to-cell signaling and interaction, carbohydrate metabolism, lipid metabolism
10	33	28	Lipid metabolism, small molecule biochemistry, molecular transport
11	31	27	Cellular development, cellular growth and proliferation, developmental disorder
12	31	27	Post-translational modification, nervous system development and function, tissue morphology
13	29	26	Organismal development, cell signaling, post-translational modification
14	29	26	Connective tissue disorders, immunological disease, inflammatory disease
15	29	26	Cell death and survival, embryonic development, nervous system development and function
16	27	25	Cellular assembly and organization, cellular function and maintenance, protein synthesis
17	27	25	Cellular movement, nervous system development and function, hereditary disorder
18	26	24	Cell signaling, tissue morphology, embryonic development
19	26	24	Gene expression, amino acid metabolism, small molecule biochemistry
20	26	24	Gene expression, cell signaling, cardiovascular system development and function
21	26	24	Cardiovascular disease, organismal injury and abnormalities, developmental disorder
22	26	24	Glutathione depletion in liver, liver fibrosis, amino acid metabolism
23	25	24	Cell-to-cell signaling and interaction, nervous system development and function, molecular transport
24	25	26	Cell morphology, post-translational modification, cellular assembly and organization
25	24	23	Behavior, organ morphology, reproductive system development and function

^a^ID represents the number of identified network, Score represents the number of genes on the network and focus molecules correspond of the number of target genes

We identified 161, 112 and 61 significative (p < 0.1) canonical pathways from the list of target genes of the bta-mir-182, bta-mir-183, and bta-mir-338, respectively (Additional file 5). From these lists, we identified five canonical pathways associated with meat tenderness regulation: apoptosis signaling, glutathione biosynthesis, regulation of cellular mechanics by calpain protease and calcium signaling and transport. Table 6 shows the miRNA and targets genes involved in these pathways.

**Table 6:** Characterization of few candidate canonical pathways for meat tenderness

Canonical Pathway	miRNA^a^	Target genes
Apoptosis signaling	bta-mir-182	*ROCK1*, *CAPN5*, *CASP2*, *MRAS*, *PRKCE*, *CYCS*, *BCL2*
	bta-mir-338	*CAPN5*
Glutathione biosynthesis	bta-mir-183	*GCLM*
Regulation of cellular mechanics by calpain protease	bta-mir-182	*CAPN5*, *GRB2*, *EZR*, MRAS, VCL, CAST
	bta-mir-183	*ITGB1*, *EZR*
	bta-mir-338	*CAPN5*
Calcium signaling	bta-mir-182	*HDAC9*, *NFAT5*, *HDAC2*, *GRIA1*, *PPP3R1*, *CREB1*, *HDAC7*, *MEF2C*, *PPP3CA*, *CAMKK2*, *GRIA3*, *PRKAR1A*
	bta-mir-183	*CAMK2D*, *ATP2C1*, *SLC8A2*, *TPM1*, *MEF2C*, *ATP2B4*
	bta-mir-338	*TP63*, *CAMK2A*, *MEF2C*, *TPM4*, *CAMK2G*
Calcium transport I	bta-mir-183	*ATP2C1*, *ATP2B4*

^a^miRNAs that exhibited the respective significative canonical pathways based on the list of target genes provided

## Discussion

Increasing evidence indicate that miRNAs play an important regulatory role in several biological processes such as cell proliferation, differentiation, neurogenesis, angiogenesis, and apoptosis as well as epigenetic changes [2, 8], which could promote phenotypic variation among individuals. Among the phenotypic traits of interest for many researchers, beef tenderness is in evidence due to economical importance to the beef industry. Thus, the present investigation was undertaken to characterize the miRNAs expressed in skeletal muscle of Nelore cattle and to identify differentially expressed miRNAs and functional pathways associated with beef tenderness in Nelore (*Bos indicus* species) cattle.

Among the miRNAs preferentially expressed in LT muscle, miR-208b and miR-499 are located within introns of myosin genes [32]; the pairs miR-1-1/133a-2, miR-1-2/133a-1, and miR-206/133b are encoded by bicistronic transcripts on different chromosomes and have been shown to play roles in the control of muscle growth and differentiation [32]. The *serum response factor* (*SRF*) and *myocyte enhancer factor*-*2* (*MEF2*) control the expression of miR-1-1/133a-2 and miR1-1–2/133a-1 [32]. These miRNAs exert opposing effects in the process mentioned above, with miR-1 playing a pro-apoptotic role and miR-133 playing an anti-apoptotic role in cardiomyocyte apoptosis [32, 33]. MiR-1 and miR-133 are evolutionary conserved and are found in most animal species, from *Drosophila* to human [9].

MiR-486 has been described as an inducer of myoblast differentiation through its negative regulation of *PAX7*, a transcription factor required for the biogenesis of muscle satellite cells and the specification of myogenic lineage [10]. MiR-206 also promotes muscle differentiation, as a previous study reported that the inhibition of this miRNA by antisense oligonucleotide inhibits cell cycle withdrawal and differentiation [34].

The differentially expressed miRNAs (bta-mir-182, bta-mir-183, and bta-mir-338) are predicted to modulate the expression of many genes that are involved in several pathways. Among the pathways identified, we chose to discuss the ones more likely to be associated with muscle proteolysis and meat tenderness.

It is widely accepted that the mechanism of meat tenderizing is an enzymatic process involving multiple proteolytic systems and that apoptosis is the first step in the conversion of muscle into meat [35]. There is large variability in meat tenderness, and this variability comes mainly from the biochemical reactions taking place during this conversion, immediately after slaughter [36].

Apoptosis is a physiological process of cell death. It is very important for development and tissue homeostasis and is mediated by a particular group of cysteine peptidases called caspases [35]. These proteins are divided according to their location in the apoptosis pathway in apoptosis initiator caspases (caspase 8, 9, 10 and 12) and effector caspases (caspases 3, 6 and 7) [37]. These enzymes play a vital role in the induction, amplification, and transduction of intracellular apoptotic signals [38] and it has been suggested that there is an overexpression of these proteases in tender meat [39].

**Fig. 4:**
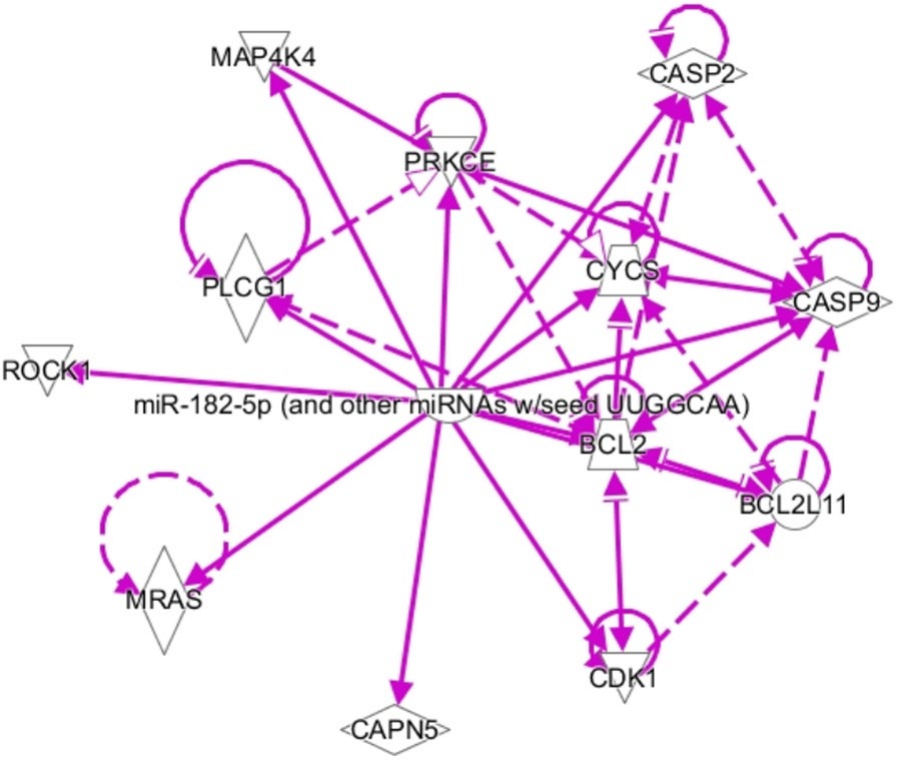
Targets molecules of bta-mir-182 involved in the apoptosis signaling pathway. Solid lines indicate a direct connection while broken lines indicate an indirect relationship

The apoptosis pathway signaling is presented in Fig. 4, where the relationship between the protein caspase and the anti-apoptotic protein BCL2 can be observed. Previous evidence report that an increase in the intracellular concentration of calcium in apoptosis results in the activation of calpain, and with this, blocking of the BCL2 protein family [40]. It has also been shown that disturbance in calcium homeostasis in the endoplasmic reticulum can lead to activation of caspase 12 by calpain and this last one can cleave the anti-apoptotic protein BCL-XL (a member of Bcl-2 family) turning it into a pro-apoptotic protein [40].

We, therefore, hypothesized that up-regulation of bta-mir-182 in the low EBVSF14 animals could be down-regulating the anti-apoptotic BCL2 protein level, and thus promoting apoptosis that could contribute to muscle proteolysis and tenderness. Conversely, it is important to note that bta-mir-182 could be downregulating *CAPN5*, *CASP2*, and *CASP9*, and this could reduce the apoptotic process.

In the apoptosis signaling pathway, it is also possible to observe the presence of *ROCK*-*1*, one of the two isoforms of *Rho*-*kinase* (*ROCK*). This protein was reported as a direct substrate of caspase-3 protein in driving the apoptosis of myocytes [41], and it also promotes actin–myosin-mediated contractile force generation by phosphorylating its target proteins. The myosin binding subunit of *myosin light chain* (*MLC*) *phosphatase 1* (*MYPT1*) *myosin light chain 2* (*MLC2*) and *LIM kinases* are downstream substrates of *ROCK*, modulating the organization of actin cytoskeleton [42].

Another important pathway identified in the study was the glutathione pathway. *Glutathione* (*GSH*) is a cysteine-containing tripeptide (glutamine, glycine, and cysteine) involved in the antioxidation system and intracellular redox state. It can be present in two forms, the reduced (*GSH*) and the oxidized glutathione (*GSSG*), and the ratio of the two forms allow the characterization of the oxidative stress in cells [43].

D’Alessandro and colleagues [44], in a study of Chianina cattle, described an accumulation of oxidative stress in tender meat samples, when *GSSG*/*GSH* ratios were higher than in tough ones. Oxidative stress might be related to meat tenderness due to the promotion of reactive oxygen species (ROS) inducing protein fragmentation [44]. The majority of *GSH* is found in the cytosol, but a small percentage is located in the mitochondria, contributing to the protection of this organelle from reactive oxygen species (ROS) [45]. Thus, the decrease of *GSH* can increase the reactive oxygen species (ROS) or accelerate the mitochondrial damage [44].

The up-regulation of bta-mir-183 in the L group could be downregulating the target gene *Glutamate*-*cysteine ligase* (*GCLM*), a rate-limiting enzyme of glutathione synthesis. The decrease in GSH can increase ROS, stimulate apoptosis and consequently contribute to tenderness.

In this context, it is important to note that in a previous study using the same animals and phenotypes used here, a QTL for SF at 24 h was found located on BTA23 at 24 Mb, regions containing the glutathione S-transferase alpha gene family [17].

Two important target genes found in our study are *calpain* and *calpastatin*. Calpain is a calcium-activated protease and injection of calcium in muscles accelerates postmortem proteolysis and tenderization [46]. In *post*-*mortem* muscle, calcium concentration in the cytoplasm increases gradually during rigor mortis while the sarcoplasmic reticulum is emptied [13]. This translocation of calcium results in different processes affecting the permeability of the sarcoplasmic reticulum membrane as binding of pro-apoptotic *Bcl2* members [40]. Calpastatin (*CAST*) is a calpain proteolytic enzyme inhibitor. Calpain’s role in *post mortem* transformation of muscle in meat is extensively studied and is widely accepted that the proteolytic activity of this protein contributes to tenderness [47, 48]. Previous studies showed that the correlations between different tenderness rates in beef, pork, and lamb are inversely related to the calpain: calpastatin ratio [49].

*Calpastatin* is a predicted target for bta-mir-182, suggesting that high expression levels of this miRNA would be reducing the translation of the *CAST* gene, resulting in a lower inhibitory effect on calpain, higher *post*-*mortem* proteolytic activity and consequently greater tenderness (Fig. 4).

Proteins of the heat shock protein family (*HSP27* and *HSP70*) were found in this study as targets of bta-mir-338 differentially expressed miRNA. Our results corroborate previous research from our group with the same animals of this work, where both proteins (*HSP70* e *HSP27*) appeared as down-regulated in animals with lower values for shear force [18]. Others authors also reported low levels of *HSP27* and HSP70-1A/B associated with animals with more tender meat [50, 51]. This evidenced negative relationship between certain *HSP* levels and meat tenderness could be linked to the anti-apoptotic activity of these proteins [47].

Regarding the common targets identified for the three differentially expressed miRNAs, we highlight the *myocyte*-*specific enhancer factor 2C* (*MEF2C*), *mitogen*-*activated protein kinase kinase kinase 2* (*MAP3K2*), and *metadherin* (*MTDH*). The *MEF2C* transcription factor is restricted to skeletal muscle, brain, and spleen, playing a crucial role in the morphogenesis and myogenesis. A previous study identified genetic variants of this gene in seven different breeds of cattle, such as, Aberdeen Angus, Charolais, Hereford, Limousin, Simmental, Polish Friesian and Polish Red, which could constitute as potential genetic markers for the characteristics of carcass and meat quality in cattle [52]. Besides that, the *MEF2C* bovine gene has been mapped on chromosome 7, which contains quantitative trait loci (QTL) responsible for the average daily gain, body and carcass weight [53] as well as the fat thickness in the *Longissimus* muscle [54]. Both the biological importance and the chromosomal location suggests that the bovine MEF2C gene could be a promising functional and positional candidate gene responsible for carcass and meat quality traits in cattle [55].

The MAP3K2 gene encodes a member of the serine/threonine protein kinase family that preferentially mediates the activation of other kinases involved in MAP kinase signaling pathway [56]. MAP3K2 is involved in cell proliferation, differentiation, and cell migration [57]. Previous studies have reported that MAP3K2 can promote cell proliferation in different types of cancer [58–60] and that some miRNAs can suppress the tumor by targeting MAP3K2 [60–62]. On the other hands, the overexpression of MAP3K2 inhibited cell proliferation in chickens but did not induce apoptosis [63]. The association of the MAP3K2 markers with loin muscle area (LMA) was previously identified in a Duroc pig population, evaluating QTL for carcass merit and meat quality traits [64], suggesting that this gene could be considered suitable candidates for future studies of growth traits and meat production in domestic animals including cattle.

The MTDH gene was also identified in a previous study, as a target gene for microRNA bta-mir-885, which was exclusively expressed in the semitendinosus muscle (STD) from Japanese black cattle when compared to masseter muscle (MS). The functional annotation of MTDH gene revealed its possible relationship in skeletal system development and regulation of transcription, respectively [65].

## Conclusion

The results obtained indicate the importance of miRNAs in the regulatory mechanisms that influence muscle proteolysis and meat tenderness and contribute to our better understanding of the role of miRNAs in biological processes associated with beef tenderness. Further studies are necessary to explore the implementation of these miRNAs as biomarkers in cattle breeding contributing to the selection of animals with improved meat tenderness.

## Additional files

**Additional file 1.** The number of raw-reads, number of reads after cleaning (filtered), number and percentage of mapped reads for High and Low groups based on estimated breeding values for shear force.

**Additional file 2.** Novel miRNAs.

**Additional file 3.** Bovine target genes of bta-mir-182 (sheet 1), bta-mir-183 (sheet 2), and bta-mir-338 (sheet 3).

**Additional file 4.** List of *Longissimus thoracis* muscle expressed genes in Nelore.

**Additional file 5.** Significative (p < 0.1) canonical pathways from the list of target genes of bta-mir-182 (Sheet 1), bta-mir-183 (Sheet 2) and bta-mir-338 (Sheet 3).

## Abbreviations

EBV: estimated breeding value
EBVSF14: estimated breeding value for shear force at 14 days
FDR: false discovery rate
H: high
IPA: ingenuity pathway analysis
L: low
LT: *longissimus thoracis*
NT: nucleotide
QTL: quantitative trait loci
