# Supplementary material for: MiRNAs differentially expressed in skeletal muscle of animals with divergent estimated breeding values for beef tenderness
Source: BMC Mol Biol. 2019 Jan 3;20:1. doi: 10.1186/s12867-018-0118-3 (PMC6317189; doi:10.1186/s12867-018-0118-3)
Supplement: Supplementary file 1 — Additional file 1. The number of raw-reads, number of reads after cleaning (filtered), number and percentage of mapped reads for High and Low groups based on estimated breeding values for shear force. [file 12867_2018_118_MOESM1_ESM.docx]

### Additional file 1. The number of raw-reads, number of reads after cleaning (filtered), number and percentage of mapped reads for High and Low groups based on estimated breeding values for shear force.

| **AnimalID** | **Raw reads** | **Filtered reads** | **Mapped reads** | **Mapped reads %** |
| --- | --- | --- | --- | --- |
| High_1_ | 953,026 | 778,341 | 734,269 | 94.3% |
| High_2_ | 2,017,539 | 1,895,184 | 1,696,375 | 89.5% |
| High_3_ | 853,194 | 612,761 | 573,292 | 93.6% |
| High_4_ | 1,827,035 | 1,629,869 | 1,330,515 | 81.6% |
| High_5_ | 1,070,514 | 892,678 | 778,134 | 87.2% |
| High_6_ | 732,192 | 509,179 | 476,628 | 93.6% |
| High_7_ | 1,342,653 | 1,205,165 | 980,711 | 81.4% |
| High_8_ | 744,149 | 623,300 | 557,099 | 89.4% |
| High_9_ | 722,385 | 598,129 | 541,383 | 90.5% |
| High_10_ | 944,644 | 633,264 | 591,842 | 93.5% |
| High_11_ | 1,467,484 | 1,309,323 | 1,119,708 | 85.5% |
| High_12_ | 1,121,000 | 974,891 | 865,371 | 88.8% |
| High_13_ | 819,280 | 589,266 | 557,088 | 94.5% |
| High_14_ | 1,387,823 | 987,356 | 926,928 | 93.9% |
| High_15_ | 791,195 | 661,599 | 586,801 | 88.7% |
| Low_1_ | 1,276,092 | 1,099,799 | 912,271 | 82.9% |
| Low_2_ | 1,358,702 | 1,181,691 | 976,534 | 82.6% |
| Low_3_ | 1,594,597 | 1,440,653 | 1,234,234 | 85.7% |
| Low_4_ | 1,520,288 | 1,366,208 | 1,215,800 | 89.0% |
| Low_5_ | 1,807,354 | 1,642,621 | 1,369,962 | 83.4% |
| Low_6_ | 1,017,064 | 632,713 | 587,320 | 92.8% |
| Low_7_ | 1,412,750 | 1,277,128 | 1,119,217 | 87.6% |
| Low_8_ | 1,607,153 | 1,443,571 | 1,216,446 | 84.3% |
| Low_9_ | 1,289,060 | 1,126,735 | 943,392 | 83.7% |
| Low_10_ | 752,934 | 471,481 | 432,670 | 91.8% |
| Low_11_ | 1,078,176 | 958,835 | 780,360 | 81.4% |
| Low_12_ | 1,834,375 | 1,251,970 | 1,177,333 | 94.0% |
| Low_13_ | 829,497 | 498,047 | 462,769 | 92.9% |
| Low_14_ | 1,140,784 | 956,047 | 705,615 | 73.8% |
| Low_15_ | 794,788 | 668,662 | 588,889 | 88.1% |
| Low_16_ | 1,289,318 | 1,129,918 | 1,014,378 | 89.8% |
| Low_17_ | 949,439 | 771,087 | 574,154 | 74.5% |
| Low_18_ | 780,269 | 555,033 | 516,077 | 93.0% |
| Low_19_ | 1,386,462 | 849,640 | 794,854 | 93.6% |
| **Mean High** | 1,119,608 | 926,687 | 821,076 | 88.6% |
| **Mean Low** | 1,248,374 | 1,016,939 | 874,857 | 86.0% |
