# Supplementary material for: MiRNAs differentially expressed in skeletal muscle of animals with divergent estimated breeding values for beef tenderness
Source: BMC Mol Biol. 2019 Jan 3;20:1. doi: 10.1186/s12867-018-0118-3 (PMC6317189; doi:10.1186/s12867-018-0118-3)
Supplement: Supplementary file 2 — Additional file 2. Novel miRNAs. [file 12867_2018_118_MOESM2_ESM.docx]

### Additional file 2. Novel miRNAs

| Provisional ID | miRDeep2 score | True positive probability | Total read count | miRNA with the same seed | Mature sequence | Genome Location - Strand |
| --- | --- | --- | --- | --- | --- | --- |
| 10_364 | 282,9 | 93 +/- 6% | 553 | - | aaaacccgaaugaacuuuuugg | 10:71809965..71810024:+ |
| 29_8151 | 178,6 | 93 +/- 6% | 348 | mmu-miR-7222-3p | cccaggacccggagcaggcugcgc | 29:50229323..50229400:- |
| 28_7720 | 150 | 93 +/- 6% | 285 | hsa-miR-6732-3p | aaacccugaacgaacuuuuugg | 28:33517923..33517979:- |
| 8_13333 | 79,3 | 93 +/- 6% | 148 | ssc-miR-7140-5p | aaacucaaacaaacuuuuuggu | 8:44112462..44112523:- |
| 1_4945 | 68,2 | 93 +/- 6% | 132 | - | aaaaaaguuuguuuggauuuuu | 1:84791929..84791990:- |
| 13_1835 | 39,2 | 93 +/- 6% | 68 | hsa-miR-548s | uuggccaaaaaguucggguuuu | 13:35041441..35041500:- |
| 17_3061 | 27,5 | 93 +/- 6% | 44 | hsa-miR-6815-3p | uggcuucuuuggaacauggcgg | 17:69000209..69000269:+ |
| 4_11177 | 21,1 | 93 +/- 6% | 39 | - | acacgcguccuuggauccugacu | 4:119142850..119142911:+ |
| 21_5320 | 19,5 | 93 +/- 6% | 29 | hsa-miR-676-3p | uuguccuacuucucagcugucu | 21:173770..173829:+ |
| 13_1712 | 19,5 | 93 +/- 6% | 29 | hsa-miR-363-5p | uggguggauggagcagcacugccu | 13:64895337..64895396:+ |
| 8_13133 | 18,6 | 93 +/- 6% | 34 | - | caaaaaguuugcuuggguuuuu | 8:38069304..38069366:+ |
| 3_10543 | 17,2 | 93 +/- 6% | 24 | hsa-miR-637 | acuggggguugagaaugucgcu | 3:19127468..19127551:- |
| 26_7237 | 14,3 | 93 +/- 6% | 18 | hsa-miR-203b-5p | caguggucucugcugagagccu | 26:34339061..34339116:- |
| 26_7098 | 13,2 | 93 +/- 6% | 18 | hsa-miR-4269 | acaggcacggccaguuugagc | 26:33108886..33108944:+ |
| 1_5026 | 11,9 | 93 +/- 6% | 20 | - | caccuagugcauggucuugggc | 1:119337665..119337722:- |
| 10_736 | 9,3 | 93 +/- 6% | 16 | - | aaacuugaaugaaccuuuuggc | 10:101213571..101213632:- |
| 12_1319 | 8,7 | 94 +/- 5% | 16 | - | acaaccugaaugaacauuuugg | 12:65685591..65685647:+ |
| 13_1627 | 8,2 | 94 +/- 5% | 14 | - | ggccacaaaguucguuuggguuu | 13:35041441..35041504:+ |
| 19_4077 | 8,1 | 94 +/- 5% | 19 | - | uuggcuagggaggaaugcugaac | 19:22852509..22852575:- |
| 16_2804 | 7,9 | 94 +/- 5% | 15 | - | acucgaacgaauuuuuggcca | 16:39279771..39279833:- |
| 15_2516 | 7,7 | 94 +/- 5% | 5 | eca-miR-9161 | ccgggccugggccgagcccca | 15:36372561..36372621:- |
| 7_12438 | 6,8 | 94 +/- 5% | 5 | mmu-miR-7240-5p | cuggagagcggaggauguaga | 7:8736592..8736674:+ |
| 12_1294 | 6,3 | 94 +/- 5% | 11 | mmu-miR-7656-3p | ccaggcuguuggaauguugacc | 12:36237559..36237618:+ |
| 6_12227 | 5,6 | 93 +/- 4% | 9 | hsa-miR-6746-5p | ucgggagacggcugacggcc | 6:120435568..120435628:+ |
| 10_691 | 5,5 | 93 +/- 4% | 45 | eca-miR-542-5p | uucggggaaggcugucagagc | 10:77217509..77217575:- |
| 5_11691 | 5,4 | 93 +/- 4% | 46 | hsa-miR-4733-3p | ccaccaggccuguggcuccgcca | 5:107074143..107074200:+ |
| 3_10734 | 5,4 | 93 +/- 4% | 12 | eca-miR-9153 | ccuccagggcuaggagguggu | 3:87838899..87838954:- |
| 22_5973 | 5,4 | 93 +/- 4% | 51 | eca-miR-191b | gaacgaaauccaagcgcagcug | 22:51947142..51947206:- |
| 11_963 | 5,3 | 93 +/- 4% | 16 | eca-miR-873 | ccaggaacugucugauugccu | 11:3086428..3086488:- |
| 10_100 | 5,3 | 93 +/- 4% | 36 | hsa-miR-7113-5p | cccagggauguagcuccuagugc | 10:20799631..20799686:+ |
| 7_12466 | 5,3 | 93 +/- 4% | 66 | mmu-miR-1950 | ucugcauccgaggacucaaccc | 7:13464152..13464212:+ |
| 20_5218 | 5,2 | 93 +/- 4% | 1301 | eca-miR-9075 | uuaggaccagaaagauggugaac | 20:7900136..7900197:- |
| 7_13029 | 5,1 | 93 +/- 4% | 8 | - | ugagaggucuguaauaacuaac | 7:83830837..83830896:- |
| 10_506 | 5 | 93 +/- 4% | 44 | eca-miR-423-5p | ugaggggcagagagugagaa | 10:13002726..13002777:- |
| 25_6958 | 5 | 93 +/- 4% | 11 | hsa-miR-30c-2-3p | uugggagaccaggggaagacu | 25:26473622..26473679:- |
| 12_1545 | 4,9 | 87 +/- 5% | 14 | hsa-miR-185-3p | aggggcugugaguccggaugauuc | 12:90653364..90653444:- |
| 18_3758 | 4,9 | 87 +/- 5% | 30 | mmu-miR-5615-3p | ugucucaguucagcaggaag | 18:61858363..61858433:- |
| 7_12693 | 4,8 | 87 +/- 5% | 17 | hsa-miR-6808-3p | cugugaccaagauacugagccu | 7:69813094..69813156:+ |
| 22_5785 | 4,2 | 87 +/- 5% | 37 | hsa-miR-345-3p | acccugaacgaacuuuuuggc | 22:37765706..37765750:+ |
| 16_2758 | 4,1 | 87 +/- 5% | 9 | hsa-miR-548d-3p | aaaaaaccgaguggacuuuuug | 16:18886162..18886220:- |
| 17_3091 | 4 | 87 +/- 5% | 8 | hsa-miR-6787-3p | ccucagcuaaaggucgaccggc | 17:75996418..75996486:+ |
| 1_5058 | 4 | 87 +/- 5% | 5 | - | ucuccaugcuguuggcucugccu | 1:148888497..148888554:- |
